# Genomic epidemiology of rifampicin ADP-ribosyltransferase (Arr) in the Bacteria domain

**DOI:** 10.1038/s41598-021-99255-3

**Published:** 2021-10-05

**Authors:** Sergio Morgado, Érica Fonseca, Ana Carolina Vicente

**Affiliations:** Laboratory of Molecular Genetics of Microorganisms, Oswaldo Cruz Institute, Rio de Janeiro, Brazil

**Keywords:** Data mining, Genome informatics, Phylogeny, Antimicrobials, Bacteria, Bacteriology, Microbial genetics

## Abstract

Arr is an ADP-ribosyltransferase enzyme primarily reported in association with rifamycin resistance, which has been used to treat tuberculosis in addition to Gram-positive infections and, recently, pan-resistant Gram-negative bacteria. The *arr* gene was initially identified on the *Mycolicibacterium smegmatis* chromosome and later on *Proteobacteria* plasmids. This scenario raised concerns on the distribution and spread of *arr*, considering the Bacteria domain. Based on 198,082 bacterial genomes/metagenomes, we performed in silico analysis, including phylogenetic reconstruction of Arr in different genomic contexts. Besides, new *arr* alleles were evaluated by in vitro analysis to assess their association with rifampin resistance phenotype. The *arr* gene was prevalent in thousands of chromosomes and in hundreds of plasmids from environmental and clinical bacteria, mainly from the phyla *Actinobacteria*, *Proteobacteria*, *Firmicutes*, and *Bacteroidetes*. Furthermore, this gene was identified in other and new genomic contexts. Interestingly, Arr sequences associated with rifampin resistance were distributed across all phylogeny, indicating that, despite the diversity, their association with rifampin resistance phenotype were maintained. In fact, we found that the key residues were highly conserved. In addition, other analyzes have raised evidence of another Arr function, which is related to guanidine metabolism. Finally, this scenario as a whole also suggested the *Actinobacteria* phylum as a potential ancestral source of *arr* within the Bacteria domain.

## Introduction

Rifampicin (rifamycins group) is a first-line antibiotic against some mycobacterial pathogens, mainly *Mycobacterium tuberculosis*. It acts binding in the β subunit of the RNA polymerase (RpoB), inhibiting the transcription initiation. Bacterial resistance against rifampicin mainly emerges by RpoB mutations, but it is also caused by enzymatic antibiotic modification, such as monooxygenation, glycosylation, phosphorylation, or ADP-ribosylation, which attenuates the rifampicin binding affinity for the RNA polymerase^1,2^. ADP-ribosyltransferases (ARTs) are a group of enzymes that catalyze ADP-ribosylation and one of them, Arr (Class I ART), inactivates rifampicin and other rifamycins. Arr is a small enzyme (16 kDa) with low sequence similarity to other known ARTs, however, shares structural homology with them. The *arr* gene was firstly described in *Mycolicibacterium smegmatis* (*arr*-ms or *arr*-1)^3^, and since then other alleles have been identified in some organisms of *Actinobacteria*, *Proteobacteria*, and *Firmicutes* phyla, in addition to marine and soil microbiomes^1,2,4,5^. However, Arr current distribution and prevalence in the Bacteria domain is unknown. The genomic context of the *arr* gene might vary by organism; for instance, while Arr-ms is chromosomally encoded in the saprophyte organism *M. smegmatis*, Arr-2 has been associated with mobilizable and mobile elements (transposons and integrons) of Gram-negative pathogenic bacteria (e.g., *Pseudomonas aeruginosa* and *Klebsiella pneumoniae*)^1^. Besides these Arr enzymes, few others (Arr-3 to Arr-8, Arr-cb, Arr-sc, Arr_Mab) have already been characterized^2,4,6–8^. Although Arr has been associated primarily with resistance to clinically important rifamycin antibiotics, some authors suggested that it may be able to act on other substrates and metabolic pathways^1,5,9^.

To date, an overview of many aspects of Arr remains poorly explored, such as its origin, transfer mechanism, epidemiology, and functionality of most of the identified alleles. Therefore, due to the impact of Arr in the clinic, its association with plasmids, occurrence in some environmental and clinical bacteria, and possible functions beyond rifamycin resistance, we performed in silico analyses to gather insights into the current distribution of Arr throughout the Bacteria domain, as well as their associated genomic contexts. We also performed in vitro analyses to assess the functionality of new *arr* alleles (carried by environmental *Mycolicibacterium* and *Mycobacteroides*) concerning resistance to rifampicin. Based on a large dataset comprising all available genomes of the RefSeq database, our analysis revealed the presence of Arr in thousands of chromosomes and hundreds of plasmids belonging to environmental and clinical bacteria, mainly from the phyla *Actinobacteria*, *Proteobacteria*, *Firmicutes*, and *Bacteroidetes*. Almost all Arr sequences presented high conservation of key residues, suggesting functionality. Furthermore, *arr* was found associated with rare (integrative and mobilizable elements) and new (prophages) genomic contexts. Interestingly, in the Arr phylogeny, functional representatives against rifamycins were observed in all clades, indicating that, despite the sequence diversity, the activity of the different Arr enzymes is conserved.

## Results

### Arr distribution and GC content

**Figure 1:**
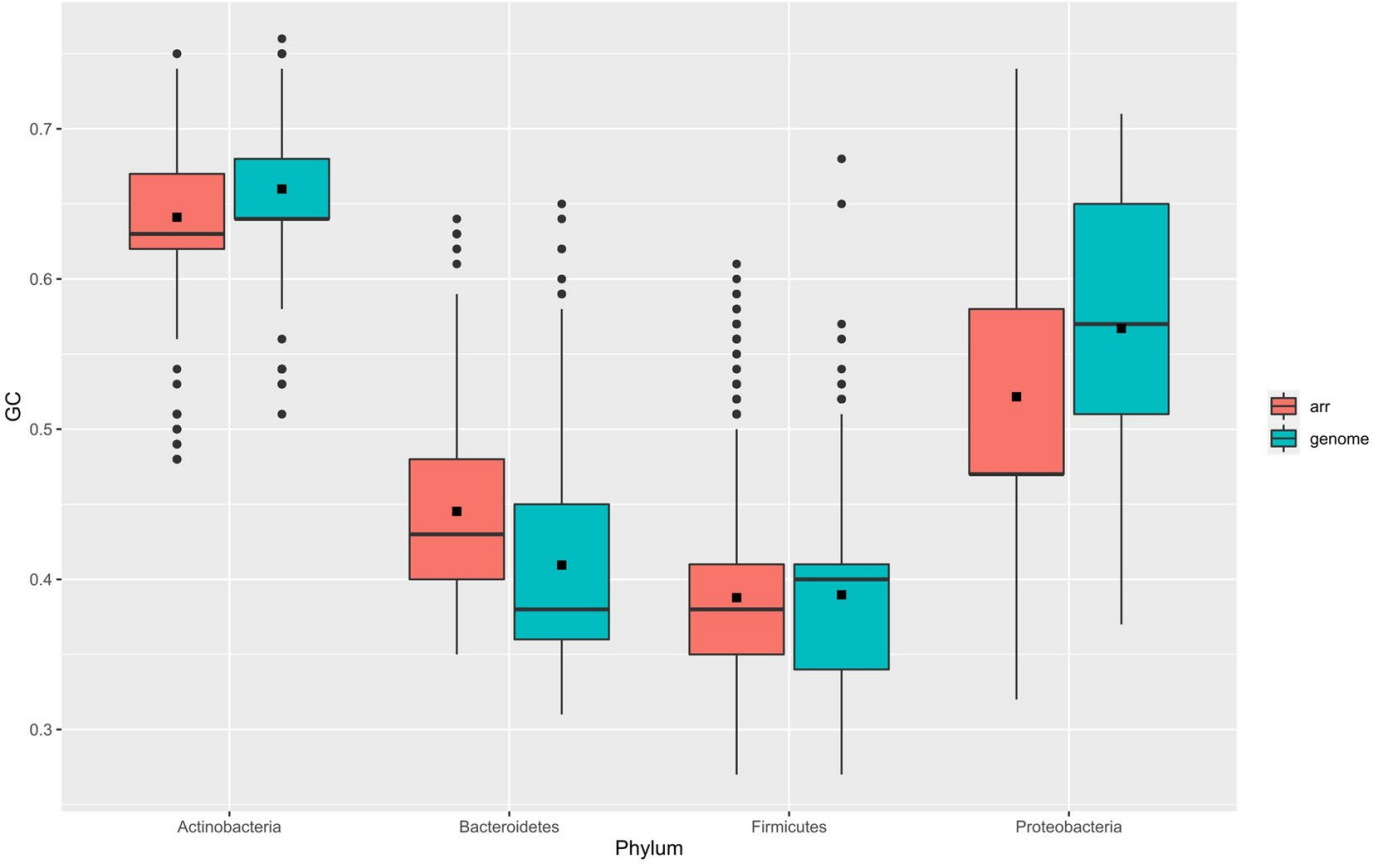
Comparison of GC content of *arr* genes and host genomes. Box plots are grouped based on the phyla with the greatest abundance of *arr*. The inner square of the boxplots corresponds to the mean value of the GC content.

Analysis of proteomes of all RefSeq available genomes (n = 198,082) revealed a narrow distribution of sequences with the ADP-ribosyltransferase domain among bacteria, being in 10,326 genomes (~ 5%). Considering the current 42 validly published bacterial phyla (https://lpsn.dsmz.de/phylum), we identified Arr in 11 phyla: *Balneolaeota*, *Ignavibacteriae*, *Rhodothermaeota*, *Deinococcus*-*Thermus*, *Verrucomicrobia*, *Acidobacteria*, *Cyanobacteria*, *Bacteroidetes*, *Firmicutes*, *Actinobacteria*, and *Proteobacteria*. Regarding the bacterial genera, Arr sequences were concentrated in *Mycobacteroides*, *Klebsiella*, *Burkholderia*, *Stenotrophomonas*, *Bacillus*, and *Escherichia* (Table S1). However, we observed that these sequences had a wide range of length (36–679 aa), in contrast to the functionally verified Arr sequences (~ 140–150 aa). Therefore, we refined the search, keeping only the sequences with at least ≥ 40% identity and ≥ 80% coverage relative to Arr-ms. This filtering resulted in the identification of 9,063 Arr sequences (Table S2) in 8,789 genomes (1–4 *arr* genes per genome) from all the aforementioned phyla, except *Ignavibacteriae*. These sequences were mainly distributed in the genomes of *Proteobacteria* (n = 3530), *Actinobacteria* (n = 3445), *Firmicutes* (n = 1387), and *Bacteroidetes* (n = 392). Among these phyla, Actinobacteria had the highest Arr relative abundance (17%) considering the phyla with more than 50 genomes (Table S3). Furthermore, analyses in lower taxonomic ranks showed higher relative abundances of Arr in classes and orders of *Actinobacteria* and *Bacteroidetes* (considering taxa with relative abundance ≥ 10% and 10 or more genomes) (Table S4 and Table S5), and in families of *Actinobacteria* (considering those with a relative abundance ≥ 20% and 10 or more genomes) (Table S6). We also observed different relative abundances of Arr considering bacterial genera (Table S7), for example, *Mycobacteroides* had 1,784 genomes and 1,774 had the *arr* gene (99%), while *Escherichia* had 21,302 genomes, but only 442 had the *arr* gene (2%). However, despite the wide distribution of *arr* within genera of various bacterial phyla, this gene has not been identified in several bacterial genera with thousands of genomes available, such as *Streptococcus* (n = 15,540), *Campylobacter* (n = 3,413), and *Neisseria* (n = 3,413). Based on these data, we verified that the *arr* gene is more likely to be species-specific. This different distribution, even within the same genus, suggests the association of *arr* to mobile elements. The species with the highest relative abundance of the *arr* gene were: *Mycobacteroides abscessus* (1675/1683 genomes), *Burkholderia cenocepacia* (321/324 genomes), *Burkholderia cepacia* (173/179 genomes), *Bacillus pumilus* (143/148 genomes), and *Staphylococcus cohnii* (79/81 genomes). However, this abundance did not mean diversity. When we analyzed the diversity of Arr sequences, filtering out the redundant ones (i.e., those with 100% identity), *Bacteroidetes* showed greater diversity (296 non-redundant Arr sequences / 401 total Arr sequences = 73%), while *Firmicutes* (41%), *Actinobacteria* (34%), and *Proteobacteria* (14%) had lower sequence diversity. A very striking example was the genus *Mycobacteroides* (*Actinobacteria*), which had the highest absolute (n = 1775) and relative (99%) Arr abundances, however, these sequences comprised only 32 non-redundant Arr sequences. This suggests that many sequences may have come from clonal bacterial genomes. The median GC content of most *arr* genes and their hosts was quite similar (Table 1). However, some phyla showed a large variation in the GC content of *arr*, which may reflect the variation in GC content within the lower taxonomic ranks of these phyla. The *arr* genes in the plasmid context also have a similar GC content relative to their hosts (Table 1). Among the phyla with the highest *arr* abundance, *Actinobacteria* had the lowest interquartile range and standard derivation of the GC content of *arr*, while *Proteobacteria* had the highest values (Table 1 and Fig. 1). This shows that *arr* genes from *Actinobacteria* have a smaller dispersion of GC content values than other phyla. In the *Firmicutes* phylum, a similar feature was also observed, however, it had more outliners and greater standard derivation of the GC content of *arr*, in addition to presenting plasmid-encoded sequences.

**Table 1:** Distribution of Arr sequences and median GC content of the *arr* and genome of their hosts.

Phylum	# Sequences	Genome median GC (%)	*arr* median GC (%)	min *arr* median GC (%)	max *arr* median GC (%)	*arr* median size (bp)	*SD of arr GC*
*Acidobacteria*	7	0.58	0.56	0.53	0.61	414	0.0336
*Actinobacteria*	3559	0.64	0.63	0.48	0.75	426	0.0388
*Bacteroidetes*	401	0.38	0.43	0.35	0.64	438	0.0620
*Balneolaeota*	1	0.48	0.38	0.38	0.38	438	–
*Cyanobacteria*	20	0.47	0.52	0.46	0.61	417	0.0340
*Deinococcus-Thermus*	2	0.65	0.55	0.5	0.61	412.5	0.0778
*Firmicutes*	1398	0.4	0.38	0.27	0.61	417	0.0604
*Proteobacteria*	3668	0.57	0.47	0.32	0.74	453	0.0687
*Rhodothermaeota*	1	0.72	0.68	0.68	0.68	417	-
*Verrucomicrobia*	6	0.6	0.59	0.55	0.65	417	0.0320
**Plasmids**							
Phylum	# Sequences	Plasmid median GC (%)	*arr* median GC (%)	min *arr* median GC (%)	max *arr* median GC (%)	*arr* median size (bp)	*SD of arr GC*
*Actinobacteria*	2	0.715	0.685	0.68	0.69	417	0.0070
*Bacteroidetes*	2	0.32	0.38	0.38	0.38	447	0
*Firmicutes*	7	0.37	0.32	0.31	0.33	417	0.00690
*Proteobacteria*	306	0.52	0.47	0.36	0.63	453	0.0368

*SD standard derivation.

### Arr phylogeny and genomic context

**Figure 2:**
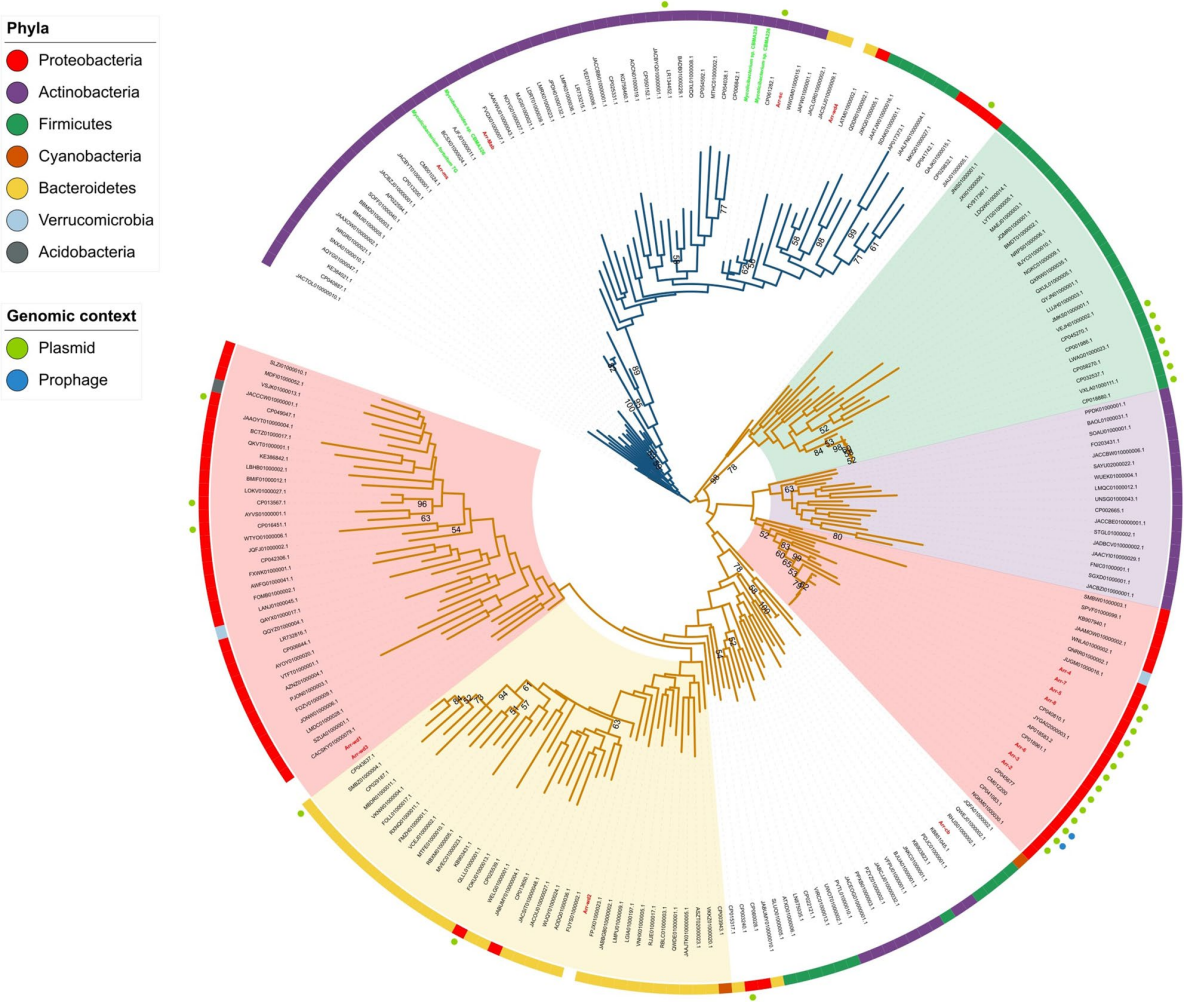
Arr phylogeny generated by the maximum likelihood method. The blue (clade I) and yellow (clade II) branches denote the two main clades. Some sub-clades of clade II are highlighted. Arr sequences functionally verified by this study and other studies are labeled green and red, respectively. Bootstrap values above 50 are shown. The outer color strips indicate the bacterial phylum of the sequence. The presence of colored circles beside the strips indicates whether the sequence has been identified in a mobile element. Reference sequences are ticked in red and have been functionally characterized.

**Figure 3:**
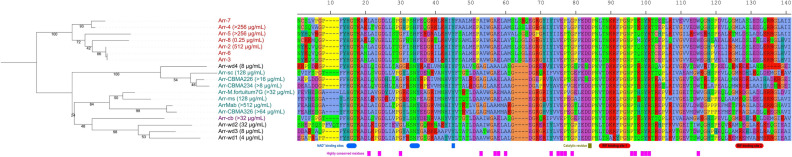
Arr phylogenetic tree and sequence alignment. The colored labels indicate the phyla of the organisms from which the sequences were obtained: red, *Proteobacteria*; aqua, *Actinobacteria*; purple, *Firmicutes*; black, environment. The value in parentheses corresponds to the MIC given by Arr expressed in heterologous systems. Highlighted residues are based on the clustalX color scheme. Conserved residues and motifs are shown below the alignment, as follows: NAD^+^ binding sites, blue; catalytic residue, olive; RIF binding sites, red. Highly conserved residues (≥ 90%), considering all Arr sequences analyzed in the phylogeny, are represented below the alignment by the fuchsia blocks. References: Arr-2, Arr-sc, and Arr-ms^1^; Arr-4 and Arr-5^6^; Arr-8^8^; Arr-w1, Arr-w2, Arr-w3, and Arr-w4^4^; ArrMab^7^; Arr-cb^2^.

To globally observe the diversity and distribution of Arr within the Bacteria domain, we constructed a phylogenetic tree with representative sequences present in mobile elements (plasmids and prophages) (n = 21) and chromosomes (n = 185). Two distinct clades were defined, depicting a divergent evolutionary pattern (Fig. 2). The clade I mainly presented sequences of *Actinobacteria*, including several genera, such as *Mycobacteroides*, *Mycolicibacterium*, *Streptomyces*, *Gordonia*, and *Arthrobacter*, and few sequences of *Bacteroidetes*, *Proteobacteria*, and *Firmicutes* phyla, suggesting an ancestral *arr* relationship with *Actinobacteria*. Interestingly, this clade contained seven sequences from *Actinobacteria* and one sequence from the environment, which were functionally verified, showing activity against rifamycins (Fig. 3). The other clade (clade II) had several sub-clades associated with specific phyla (e.g., *Proteobacteria*, *Firmicutes*, *Actinobacteria*, and *Bacteroidetes*), appearing to evolve in a more divergent manner than clade I (Fig. 2). As in clade I, along clade II there were also Arr sequences involved with resistance to rifampicin (Fig. 3). Overall, plasmid associated Arr sequences were spread across both clades along with chromosomal sequences. Particularly, *Proteobacteria* and *Firmicutes* sub-clades were related to plasmids. Considering *Proteobacteria*, two main subclades have been defined, one comprising sequences mainly from chromosomes and the other with sequences mainly from plasmids. While for *Firmicutes* most of its sequences were grouped in a single sub-clade encompassing both chromosomal and plasmid sequences (Fig. 2).

**Figure 4:**
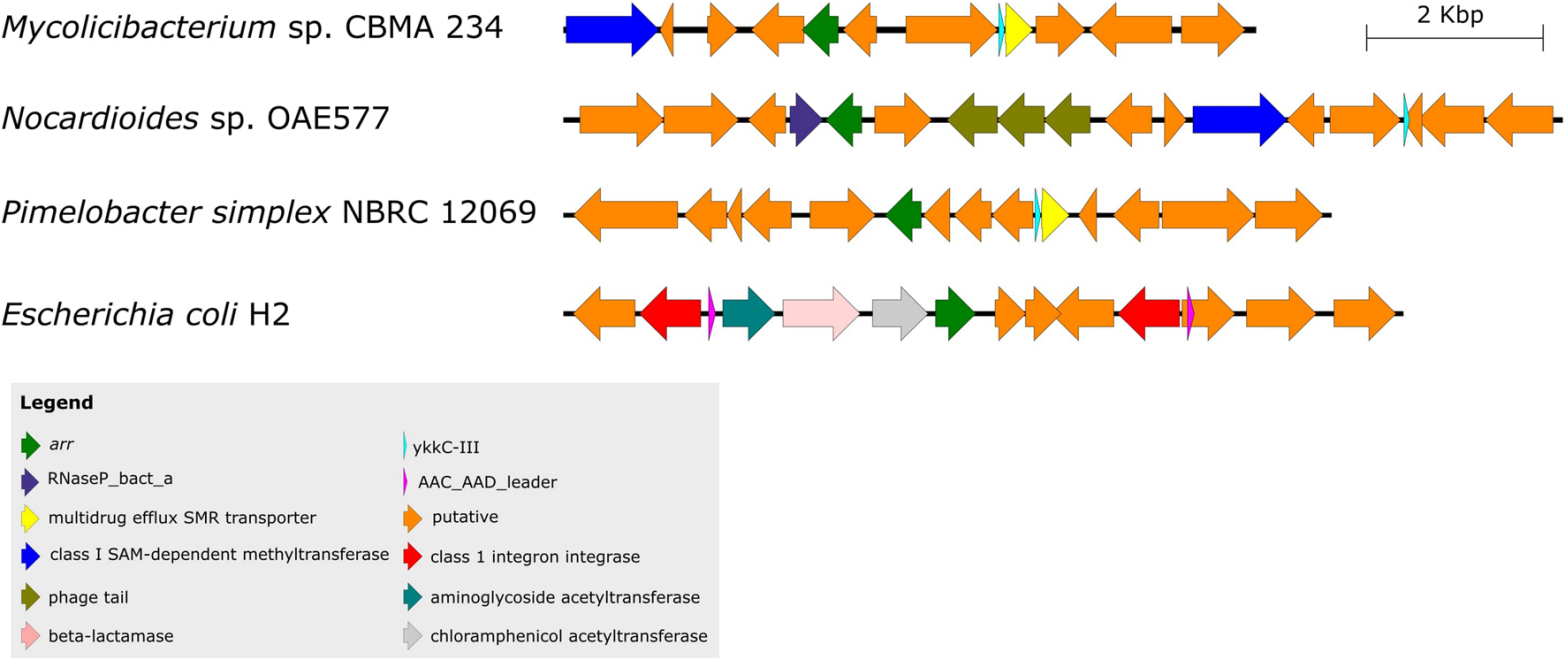
*arr* genomic context in different organisms.

In general, the genomic context analysis did not identify a universal context associated with *arr*, as well as mobile elements. However, *arr* genes in the context of integrons and transposons have been identified in several *Proteobacteria* genomes, along with other antibiotic resistance genes, such as β-lactamase, chloramphenicol, and aminoglycoside acetyltransferases; and sometimes close to the AAC_AAD_leader ncRNA (Fig. 4). In fact, ncRNAs (including riboswitches) were abundant elements in the *arr* neighborhood of several phyla, since 2,454 ncRNAs from 74 different ncRNA species were observed in 2,111 genomes (1–4 ncRNA per *arr* neighborhood). Interestingly, even in different genomic contexts of unrelated organisms, some same species of ncRNAs were present in the vicinity of *arr*. For example, among some *Actinobacteria* genomes of *Mycolicibacterium*, *Nocardioides*, and *Pimelobacter*, the *arr* gene was in distinct genomic regions only a few genes upstream/downstream the ncRNA ykkC-III and/or RNaseP_bact_a ncRNA (Fig. 4). Curiously, two *arr* genes were found in prophages associated with plasmids from *Klebsiella pneumoniae* (*Proteobacteria* phylum) (CM012200 and CP045677). Besides, two other *arr* genes were identified within regions predicted as integrative and mobilizable elements (IMEs). An IME of 6.3 kb was predicted in a contig (VSJI01000078) of *K. pneumoniae* encoding: class 1 integron integrase, *arr*, erythromycin esterase (ereA), β-lactamase (OXA-48 family class D), relaxase (mobA), in addition to two hypothetical genes. The other IME had 18.4 kb length, predicted in a contig (NUOQ01000012) of *Priestia megaterium* (*Firmicutes* phylum), and encoded 17 genes, including integrase, merR, *arr*, three relaxases (Mob_Pre, Replic_Relax, and MobA_MobL), and lysM.

We also performed a phylogenetic analysis considering only the Arr plasmid sequences to verify a strict/loose association with bacterial phyla. The analysis included 317 Arr sequences retrieved by in silico searches, in addition to functionally verified Arr sequences (Figure S1). In this analysis, it was revealed that Arr-2 to Arr-8 represent most sequences carried exclusively by *Proteobacteria* plasmids, and likewise, there are sets of Arr sequences related exclusively to *Actinobacteria* and *Firmicutes* plasmids. Therefore, in general, there is a strict association of the Arr plasmid clusters with their hosts.

### Cloning of the mycobacteria *arr* genes

In addition to in silico analysis, we experimentally tested four new chromosomal *arr* alleles (belonging to clade I of Fig. 2) identified in three *Mycolicibacterium* and one *Mycobacteroides* strains (CBAS, Bacterial Collection, Fiocruz/Brazil) to determine their relation to rifampicin resistance. All these alleles conferred resistance to rifampicin in the heterologous system, inducing higher MIC levels compared to the wild-type *Escherichia coli* (4 µg/mL). The *Mycolicibacterium* alleles induced a two to eightfold increase in MIC values (8–32 µg/mL), whereas the *Mycobacteroides* allele induced at least a 16-fold increase in MIC values (> 64 µg/mL) (Table 2). Comparatively, the activity of these cloned alleles against rifampicin was similar to that of the wild organisms (Table 2).

**Table 2:** E-test MIC values for rifampicin in *E. coli* and wild organisms.

Strains	*E. coli*-wd	*E. coli*-*arr*_CBMA226_	*E. coli*-*arr*_CBMA234_	*E. coli*-*arr*_CBMA326_	*E. coli*-*arr*_*M.fortuitum7G*_	*M. sp.* CBMA226	*M. sp.* CBMA234	*M. sp.* CBMA326	*M. fortuitum 7G*
Rifampicin MIC (µg/mL)	4	16	8	32	32	16	16	32	32

### Sequence conservation and structure analysis

**Figure 5:**
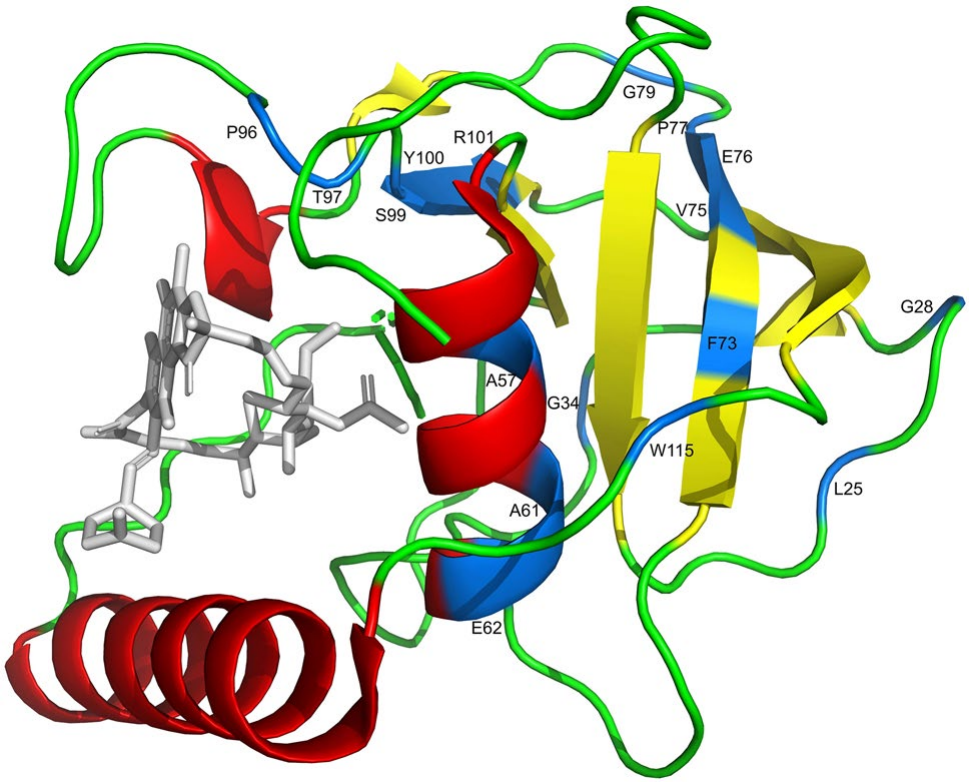
Structure of Arr-ms (PDB 2HW2) highlighting motifs (α-helices, red; β-sheets, yellow; loops, green) and the highly conserved residues identified in all Arr sequences used in the phylogeny (blue residues). Rifampin corresponds to the gray structure.

Since the different *arr* alleles conferred different MIC values, we analyzed their protein sequences concerning the conserved residues and motifs proposed to be needed for the Arr function (NAD^+^ binding sites, catalytic residue, and RIF binding sites). Among the experimentally tested Arr proteins, the NAD^+^ binding sites were highly conserved, being composed of the motifs HGT (100%) and S[NH][YF] (95% for S, 63% for N, 37% for H, 53% for Y, 47% for F) and Y45 residue (100%) (Table S8 and Fig. 3). The catalytic residue, D84, also presented 100% of conservation (Table S8 and Fig. 3). The RIF binding sites 1 (87–95 aa) and 2 (126–133 aa) of the Arr sequences encoded by these alleles showed different conservation levels, with the first site showing more conservative replacements (Table S8 and Fig. 3). Some residues from these sites were specific to the organisms analyzed, such as L87 (*Proteobacteria*) and V87 (*Actinobacteria*) at site 1; and L127 (*Proteobacteria*) and R127 (*Actinobacteria*) at site 2 (Fig. 3). We also analyzed the conservation of these key residues and motifs in all Arr sequences used in the phylogeny, and the high conservation (≥ 90%) is maintained (Table S9). In addition, we could identify dozens of other highly conserved residues (≥ 90%) along the Arr sequences: L21 (94%), G24 (97%), G30 (91%), A53 (99%), A57 (97%), E58 (90%), A60 (92%), Y73 (98%), V75 (97%), E76 (91%), P77 (93%), G79 (98%), P96 (96%), T97 (96%), S99 (99%), Y100 (92%), R101 (100%), and W115 (98%) (Fig. 3, fuchsia blocks; and Table S9). Since these residues are highly conserved, they could also be essential to the structure of the Arr. So, we mapped these residues in the X-ray crystal structure of Arr-ms (PDB 2HW2), observing that most of them are near of β-sheets or positioned on the α1-helix, and on the β4 and β6 sheets (Fig. 5).

## Discussion

The phenomenon of antibiotic resistance has been widely explored in the clinical context. However, it is becoming quite clear that natural evolutionary forces modulate this phenomenon in environmental bacteria, later impacting the clinic. Here, based on a comprehensive genomic investigation, an overview of the *arr* epidemiology, transfer mechanism, origin, and functionality of new alleles in the bacterial domain was raised. Our analysis indicated that Arr has evolved in different patterns in a wide spectrum of bacteria, revealing its distribution in thousands of bacterial genomes of at least ten phyla, particularly in *Actinobacteria*, *Proteobacteria*, *Firmicutes*, and *Bacteroidetes*. Regarding plasmid born Arr, sequence diversity was also observed, however, there was a conservative pattern within each phylum of bacteria, suggesting that in some cases there was a likely amelioration process^10,11^. Interestingly, one of the Arr *Proteobacteria* clades, mainly formed by Arr encoded in clinical pathogenic *Proteobacteria* plasmids, has a phylogenetic relationship with a clade that encompasses only chromosomal Arr of environmental *Actinobacteria*, which suggests, in this case, the environment as a source of Arr currently impacting the clinic. This scenario substantially enlarged the occurrence and distribution of Arr within the Bacteria domain, since the few previous studies on this issue considered a restricted set of bacteria carrying Arr^1,4^.

Arr belongs to class I ARTs, which contain the highly conserved H-Y-[QED] motif^1,12^. However, only using structure-based sequence alignment is possible identify the residues H19 (H15 in Fig. 3) and Y49 (Y45 in Fig. 3) in Arr-ms^1^. The glutamic acid residue of this motif, which is a critical catalytic residue in other ARTs, such as diphtheria toxin and PARP-1^13^, is replaced in Arr by an aspartate residue (D84 in Fig. 3)^1,14^. Here, we observed that this H-Y-[QED] motif is highly conserved in almost all hundreds of Arr sequences analyzed, indicating its importance in Arr activity, and suggesting that these Arr sequences could be functional. Indeed, the Arr enzymes already functionally characterized (Arr-ms, Arr-2 to Arr-8, Arr-cb, Arr-sc, Arr_Mab, Arr-wd1-4)^2,4,6–8^ and those of the present study were observed along the phylogenetic tree, showing that despite the sequence diversity, even in the RIF binding sites, their activities against rifamycins are maintained. These patterns were observed in sequences carried by bacteria belonging to high and low antibiotic-impacted environments. In addition, other highly conserved residues were identified along the Arr sequences, which could constitute sites with impact on Arr function in relation to rifamycins or other substrates. Indeed, some of these residues were positioned in one α-helix and two β-sheets, including α1-helix and β6-sheet, which make contact with NAD^+^ and rifampin^1^. Despite these evidence, it is not possible to conclude that all Arr proteins identified in the bacterial domain are active. For example, an Arr was demonstrated to be inactive, with respect to rifampin, by the Q127 → R variation, even with the conservation of the NAD^+^ binding sites and catalytic residue^2^. However, in our analysis, several variations in this residue (site 132 in Fig. 3) were observed in Arr active enzymes. Interestingly, two sequences (Arr-wd1 and Arr-wd3) from soil metagenomes that showed the R127 variation (R132 in Fig. 3) were reported as functional, but with lower MIC values compared to the other sequences. This suggests that just a specific amino acid variation might not be enough to inactivate Arr, at least to act on the modification of rifampicin, but in vitro experiments would be needed to confirm these predictions.

In general, among the phyla, the *arr* genes and their host genomes had a similar median GC content, however, in some cases, large variations of maximum and minimum GC content of *arr* were observed within a phylum, which could be evidence of transfer of this gene from unrelated organisms. In fact, *arr* genes have been found in association with a variety of genomic contexts, including mobile platforms (integron, transposon, and plasmids), particularly in *Proteobacteria*, in addition to chromosomes^1,6,15^. Here, this scenario was expanded with the identification of new and rare genomic contexts for this gene, in addition to its identification in several plasmids of *Firmicutes*, *Actinobacteria*, *Cyanobacteria*, and *Proteobacteria*. To date, few reports on the occurrence of *arr* in the context of IMEs have shown that this gene has been spread by the Salmonella Genomic Island 1 (SGI1) in *Enterobacteriaceae*^16^. Here, two new IMEs were revealed in association with *arr*, one in *Klebsiella* (Proteobacteria) and the other in the ubiquitous *Priestia megaterium* (Firmicutes). Interestingly, *arr* was also identified in a new context, in archaeological remnants of prophages, which means, at least an ancestral association with bacteriophages. Therefore, this piece of evidence on the association of *arr* with various mobile platforms and chromosomes reinforces that the widespread of *arr* in the bacterial domain is due to vertical genetic inheritance and horizontal transfer.

The wide distribution of the *arr* gene in environmental bacteria and the apparent plasticity of Arr interactions suggest that it may be able to act on substrates other than rifamycins^1,5,9^. Genomic analyses considering the different *arr* genetic neighborhoods revealed some common elements close to the *arr* gene, such as ncRNA genes (e.g., AAC_AAD_leader, RNaseP_bact_a, and ykkC-III). Some of these ncRNAs are riboswitches, suggesting that these ncRNAs could be acting, at some level, in *arr* regulation. Interestingly, ykkC-III is a member of the ykkC riboswitch family commonly found in *Actinobacteria*, and which has recently been characterized as a guanidine-specific genetic regulatory element (guanidine-III riboswitch)^17^. Among the genes regulated by ykkC-III already described, there is the small multidrug resistance (SMR) transporter gene^18^, and in fact, here some of the identified ykkC-III were adjacent to these SMR transporters. Therefore, based on the vicinity of *arr* and ykkC-III in dozens of *Actinobacteria* genomes, and that some ADP-ribosyltransferases acts on guanidino compounds^19–21^, we hypothesize that *arr* could also be associated with guanidine metabolic pathways, in addition to modulation of reactive oxygen species (ROS) and modification of rifamycins^9^.

Zhang et al. (2020) suggested *Flavobacteriaceae* as a potential ancestral source of tigecycline resistance tet(X) gene based on its high prevalence in the chromosome of organisms from this family and similar GC content^22^. Considering our findings of the high prevalence of the *arr* gene in the chromosome of several taxa of the *Actinobacteria* phylum; similar GC content between the *arr* genes and their hosts; the lowest interquartile range and standard derivation of the GC content of *arr* compared to the other phyla; we hypothesized organisms of the *Actinobacteria* phylum as one of the potential ancestral sources of *arr*.

## Methods

### Genomes analyzed and Arr identification

A collection of 198,082 bacterial genomes was obtained from the RefSeq database in October/2020 and surveyed to identify Arr sequences through protein signatures by using an HMM profile with the hmmsearch program^23^ (e-value of 1e-10). This HMM profile was built with 186 proteins from NCBI assigned as “rifampin ADP-ribosyltransferase”. The sequences identified by this HMM profile were filtered, keeping those that showed at least 40% identity and 80% coverage concerning Arr-ms (WP_011727512.1). Taxonomic data for each genome was retrieved using NCBI Entrez Direct (EDirect).

### Arr genomic compartments

The proteomes carrying Arr had their genomic data retrieved, and *arr* sequences were assigned to three genomic compartments: chromosome, plasmid, and prophage. The *arr* genes within sequences named as plasmid by the NCBI were thus considered; while the *arr* genes encompassed in regions with viral signatures, determined by the ProphET program^24^, were considered of prophage origin. The other *arr* sequences were considered of chromosomal origin. Integrative and mobilizable elements were surveyed using ICEfinder web-based tool^25^. The genomic context of *arr* genes was represented using EasyFig v2.2.5^26^.

### Phylogenetic analysis

Arr sequences showing an identity above a threshold (100% for sequences obtained from plasmids and 70% for sequences from other compartments) and ≥ 70% coverage were clustered using CD-HIT v4.7^27^ and submitted to the phylogenetic analysis. The representative Arr sequences were aligned with MAFFT v7.407^28^ in GUIDANCE2 v2.02^29^, which removed ambiguously aligned positions, and maximum-likelihood trees with 500 bootstrap replicates were built by Seaview v4.7^30^ and draw by iTOL^31^. Mega X^32^ was used to determine the best substitution model (WAG + G) and likelihood score (-34,826.278). Experimentally validated Arr sequences were used as references (Arr-ms, WP_011727512.1; Arr-2, WP_000237816.1; Arr-3, WP_001749986.1; Arr-4, WP_033959319.1; Arr-5, WP_052238312.1; Arr-6, AEU08942.1; Arr-7, WP_044059783.1; Arr-8, WP_063857695.1; Arr-sc, WP_011028626.1; Arr-cb, WP_002589901.1; Arr-MAB_0591, WP_063842202.1; Arr-wd1, Arr-wd2, Arr-wd3, Arr-wd4^4^).

### Sequence conservation and structure analysis

The alignment of all Arr sequences analyzed in the phylogeny was submitted to JProfileGrid v2.0.5^33^ to determine the conservation of the residues. Known key residues, related to NAD^+^ binding sites, catalytic residue, and RIF binding sites, were analyzed to predict the potential functionality of the Arr sequences. In addition, other highly conserved residues, which were not proposed as key residues, were mapped against a previously reported 3D structure of Arr-ms (PDB 2HW2)^1^. The 3D image was prepared with PyMol v2.3.0 software (https://pymol.org/2/).

### Bacterial strains and expression of *arr* genes in a heterologous system

Four *Mycobacteriaceae* strains were employed in this study for in vitro analyses, including three *Mycolicibacterium* (*Mycolicibacterium* sp. CBMA226, *Mycolicibacterium* sp. CBMA234, and *Mycolicibacterium* fortuitum 7G) and one *Mycobacteroides* (*Mycobacteroides* sp. CBMA326). They were isolated from Atlantic Forest soil and deposited in the Bacteria Collection of Environment and Health (CBAS, Fiocruz Institute-Brazil). These strains were grown in tryptic soy broth (TSB) agar plates for six days at 23 °C.

The *arr* gene was amplified using specific primers for each strain: *M.* sp. CBMA226 (5’-GGGACAGCACAATTCGATC-3’ and 5’-TGACGTTCTTCTGGAGGACA-3’), *M.* sp. CBMA234 (5’-AGCATCGCTGAGTTCAAGG-3’ and 5’-TTAGCTGTTTGACCCTGCTG-3’), *M. fortuitum* 7G (5’-CGCTGTTCCCACTCTCACTA-3’ and 5’-CACAAATCCCCGTATCGAG-3’), and *M.* sp. CBMA326 (5’-GAGATTTGTAGCGGCATGAG-3’ and 5’-TGGTGATCTTCGTTGGACTC-3’). The PCR products comprising the entire open reading frame of the *arr* alleles were Sanger sequenced and deposited in GenBank (arr-M.fortuitum7G, OK086685; arr-CBMA226, OK086686; arr-CBMA234, OK086688; arr-CBMA326, OK086687). Then, the PCR products were cloned into the pGEM T-Easy Cloning Vector System (Promega), used to transform competent *E. coli* DH5α lineage, and plated onto LB containing ampicillin 100 µg/mL and rifampicin 6 µg/mL. The transformant DNA was obtained and used as a template in PCR reactions performed to determine the presence and orientation of the insert (*arr* gene), related to P_lac_ promoter, by using primers targeting the insert cloning site provided by the kit. After confirming the position of the cloned *arr*, the rifampicin MIC of the transformants was determined by the E-test method (bioMerieux) in Mueller–Hinton agar plates, in triplicates, according to CLSI guidelines. As a control, the *E. coli* DH5α presented rifampicin MIC of 4 µg/mL.

## Supplementary Information

Supplementary Information 1.

Supplementary Information 2.
