# Supplementary material for: Genomic epidemiology of rifampicin ADP-ribosyltransferase (Arr) in the Bacteria domain
Source: Sci Rep. 2021 Oct 5;11:19775. doi: 10.1038/s41598-021-99255-3 (PMC8492726; doi:10.1038/s41598-021-99255-3)
Supplement: Supplementary file 1 — Supplementary Information 1. [file 41598_2021_99255_MOESM1_ESM.pdf]

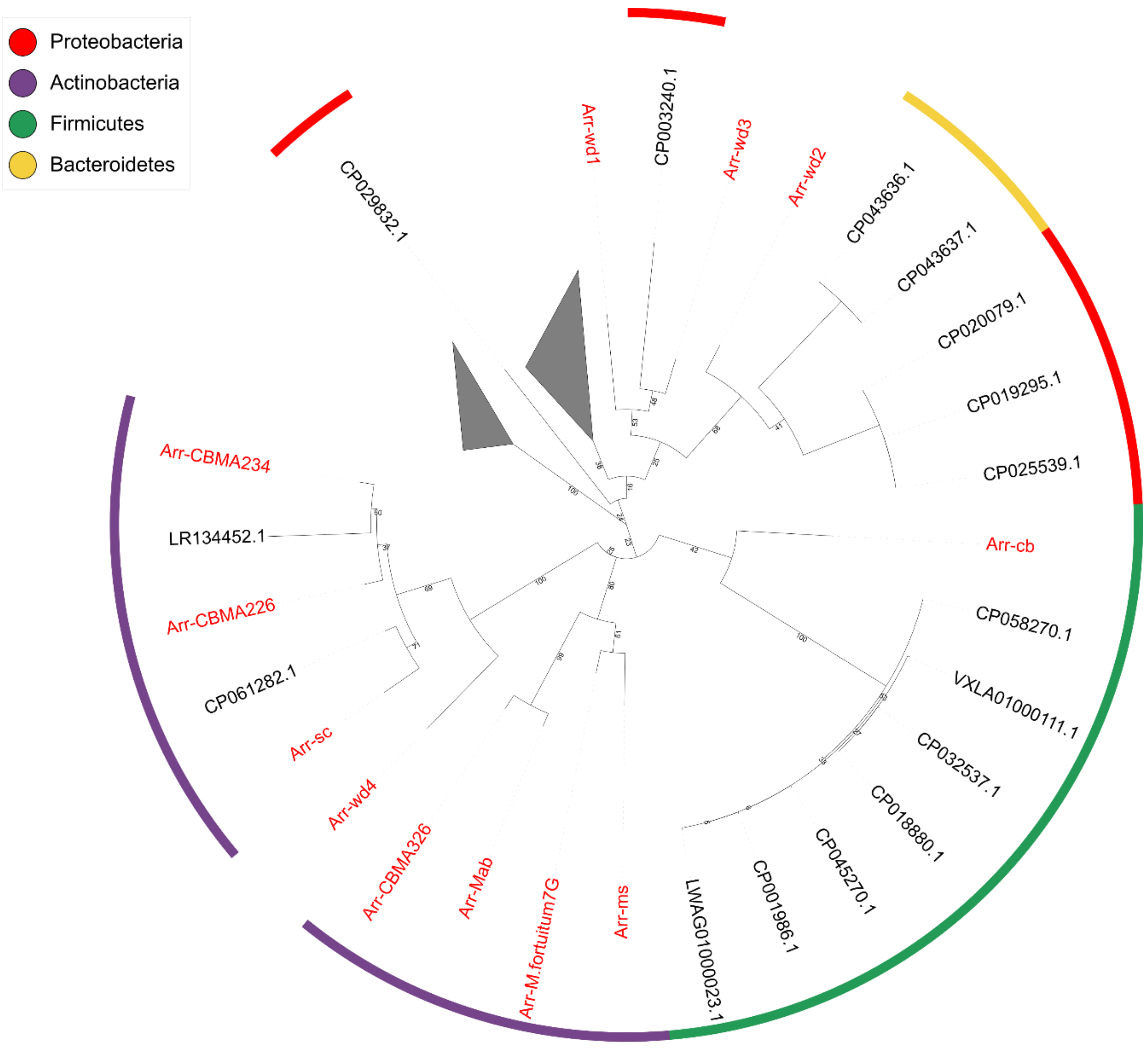

Figure S1: Plasmid Arr phylogeny generated by the maximum likelihood method. Two *Proteobacteria* clusters were collapsed, one with 273 sequences (including Arr-2 to Arr-8), and the other with 35 sequences. Bootstrap values above 50 are shown. The reference sequences are labelled in red and have been functionally characterized.
